# Beyond caregiving: Navigating life with a developmentally disabled daughter with a severe case of Hidradenitis Suppurativa

**DOI:** 10.1002/ski2.403

**Published:** 2024-06-04

**Authors:** Elisha M. Myers, Janelle S. Nassim

**Affiliations:** ^1^ Trinity College Dublin Dublin Ireland; ^2^ Charles E. Schmidt College of Medicine Florida Atlantic University Boca Raton Florida USA; ^3^ Department of Dermatology Indiana University School of Medicine Indianapolis Indiana USA

## 1 CAREGIVERS PERSPECTIVE

I've been my daughter's full‐time caretaker since she was born, and now she's in her 50s. When she was born, she didn't get enough oxygen to her brain, which caused her to have learning difficulties. It's just been her and me living together at home.

At first, we thought she had acne when she was 18 because of some boils on her face, but it kept getting worse. We went to a private dermatologist who gave her antibiotics and isotretinoin, but none of the medications were able to help her. The boils didn't stop on her face; they spread to her groin, buttocks, and back. Over the years, we went to many doctors, trying different antibiotics and injections, but nothing worked. After more than 5 years, she was finally diagnosed with Hidradenitis Suppurativa (HS).

Her life truly changed overnight. She became housebound, unable to even walk at times because of the boils between her legs. She used to enjoy meeting friends for shopping or going to the caravan on weekends, but all of that stopped as the HS worsened. I remember her crying, telling me that she felt like she was a ‘burden’, but I always told her she wasn't. She was good at hiding her pain, always saying she was alright. I tried to keep my mindset positive, not focussing on the negatives, but my life changed significantly too. I was no longer able to work, something I had always enjoyed. Instead, we were forced to rely completely on government assistance, something that caused me significant stress. I could no longer see family and friends often, missing family holidays as I was not willing to leave her side. I spent hours trying my best to help her, doing research online and asking people in other countries what they used to treat HS.

She had to have seven operations for the boils under her arms and on her thighs. I felt like they were cutting away all of her body, and right after the operation it always felt like a new boil would come up to replace the one they had just removed. After one of the operations while she was in the hospital, she got MRSA, and every time we went to the hospital after that she was unable to go to a surgical ward even if she tested negative. It felt like a never‐ending cycle, and the doctors told us it was one of the worst cases they had ever known.

The doctors and nurses we worked with were excellent. They kept trying even when nothing seemed to work, and they were always kind to her. We had nurses come to our home who came every day, often twice a day, to change her bandages and keep her wounds clean. They also spent time teaching me how to change the bandages so I could do it when they were not there. I remember how badly the boils smelled when we changed the bandages. You would lift up the bandages, and pus would go everywhere; it was awful. Even when there was no bacteria, the smell was still there. Whilst at home and in the hospital, her bedding had to be changed daily, with the hospital staff sterilising her room every day. She used to say to the doctors and nurses that she was not dirty and was always very worried in case they thought she was.

The journey to finding a medication that worked took over 20 years. Her doctors prescribed her Adalimumab and steroid injections, which have been working well. Before this, I often was unable to sleep. I remember multiple nights when she was so ill, I worried she would die. I would lay in bed wide awake, feeling completely hopeless. I do often still worry that the boils will come back but am hopeful the medications will continue to work and new medications will be developed to treat HS. Although she no longer has the boils, she still has scars from her many operations, but I feel lucky that she doesn't have any on her face. Although it was a really hard time for both of us, we really tried to make the best of it. To others going through this, I would say they should remain hopeful, take it day by day, and not be afraid to ask for help if needed.

## 2 MEDICAL PERSPECTIVE

Hidradenitis suppurativa (HS) is a chronic inflammatory skin condition, characterised by painful nodules and abscesses under the skin.^1^ HS imposes a significant morbidity on patients lives, including physical discomfort, emotional distress, social isolation, and economic challenges. Furthermore, the recurrent nature of flare‐ups contributes to a sense of frustration and helplessness among patients, affecting their overall quality of life.^2^

Emotionally, HS takes a profound toll, leading to feelings of shame, embarrassment, and low self‐esteem.^3^ Coping with the visible manifestations of the disease and the uncertainty of its progression can exacerbate anxiety and depression, further isolating individuals from social interactions and support networks. Socially, HS patients often face stigma and discrimination due to the visible nature of their condition, leading to social withdrawal and avoidance of public situations.^4^ This social isolation can perpetuate feelings of loneliness and alienation, exacerbating the psychological burden of the disease. Economically, managing HS can be financially draining, with expenses related to medical consultations, treatments, and medications quickly accumulating.^5^ Moreover, the impact of HS on employment and productivity may lead to loss of income and economic instability for affected individuals and their families.^5^

Treatment for HS is dependent on disease severity and usually involves a multifaceted approach, combining the use of both medications and procedural interventions. Medications include oral antibiotics like tetracyclines, topical therapies, biologic therapies, and hormonal therapies.^6^ Surgical options, along with lifestyle modifications like weight loss and smoking cessation, can also be used. HS treatments continue to evolve, with newer medications targeting the IL‐17 and janus kinase‐signal transducer and activator of transcription (JAK‐STAT) pathways,^7^ providing hope for patients with treatment resistant HS.

In conclusion, HS presents a multifaceted onus on patients, impacting various aspects of their lives. Recognising and addressing these burdens is important for healthcare providers to deliver comprehensive care and support, improve treatment outcomes, and enhance the overall well‐being of individuals living with HS.

## AUTHOR CONTRIBUTIONS

**Elisha M. Myers**: Conceptualization (equal); data curation (equal); formal analysis (equal); funding acquisition (equal); investigation (equal); methodology (equal); project administration (equal); resources (equal); software (equal); supervision (equal); validation (equal); visualization (equal); writing—original draft (lead); writing—review and editing (equal). **Janelle S. Nassim**: Conceptualization (equal); data curation (equal); formal analysis (equal); funding acquisition (equal); investigation (equal); methodology (equal); project administration (equal); resources (equal); software (equal); supervision (equal); validation (equal); visualization (equal); writing—original draft (supporting); writing—review and editing (equal).

## CONFLICT OF INTEREST STATEMENT

None to declare.

## ETHICS STATEMENT

Not applicable.

## PATIENT CONSENT

Caregiver's written consent for paper publication was obtained.

## Data Availability

Data sharing not applicable to this article as no datasets were generated or analyzed during the current study.
